# Comparison of Blood Flow Restriction Training versus Non-Occlusive Training in Patients with Anterior Cruciate Ligament Reconstruction or Knee Osteoarthritis: A Systematic Review

**DOI:** 10.3390/jcm10010068

**Published:** 2020-12-27

**Authors:** Cristina Bobes Álvarez, Paloma Issa-Khozouz Santamaría, Rubén Fernández-Matías, Daniel Pecos-Martín, Alexander Achalandabaso-Ochoa, Samuel Fernández-Carnero, Antonio Martínez-Amat, Tomás Gallego-Izquierdo

**Affiliations:** 1 Physiotherapy Center, Alcalá de Henares University, 28801 Madrid, Spain; crbobes@gmail.com (C.B.Á.); palomaiks@gmail.com (P.I.-K.S.); 2 Clínica Fisiogijón, 33213 Gijón, Spain; 3 Hospital Universitario Quironsalud, 28223 Pozuelo de Alarcón, Spain; 4 Research Institute of Physiotherapy and Pain, Alcalá de Henares University, 28801 Madrid, Spain; ruben.fernanmat@gmail.com (R.F.-M.); daniel.pecos@uah.es (D.P.-M.); samuel.fernandezc@uah.es (S.F.-C.); tomas.gallego@uah.es (T.G.-I.); 5 Department of Physiotherapy and Nursing, Alcalá University, 28871 Alcalá de Henares, Spain; 6 Department of Health Sciences, Universidad de Jaén, 23071 Andalucía, Spain; amamat@ujaen.es

**Keywords:** blood flow restriction, blood flow restricted training, kaatsu, occlusion training, blood flow occlusive, anterior cruciate ligament reconstruction, knee osteoarthritis, blood flow restriction (BFR), knee

## Abstract

Patients undergoing anterior cruciate ligament (ACL) reconstruction and patients suffering from knee osteoarthritis (KOA) have been shown to have quadriceps muscle weakness and/or atrophy in common. The physiological mechanisms of blood flow restriction (BFR) training could facilitate muscle hypertrophy. The purpose of this systematic review is to investigate the effects of BFR training on quadriceps cross-sectional area (CSA), pain perception, function and quality of life on these patients compared to a non-BFR training. A literature research was performed using Web of Science, PEDro, Scopus, MEDLINE, Dialnet, CINAHL and The Cochrane Library databases. The main inclusion criteria were that papers were English or Spanish language reports of randomized controlled trials involving patients with ACL reconstruction or suffering from KOA. The initial research identified 159 publications from all databases; 10 articles were finally included. The search was conducted from April to June 2020. Four of these studies found a significant improvement in strength. A significant increase in CSA was found in two studies. Pain significantly improved in four studies and only one study showed a significant improvement in functionality/quality of life. Low-load training with BFR may be an effective option treatment for increasing quadriceps strength and CSA, but more research is needed.

## 1. Introduction

Blood flow restriction (BFR) or Kaatsu, consists of partially restricting the arterial inflow and completely restricting the venous return flow during exercise [1]. Restrictive straps or a blood pressure cuff are placed on the proximal area of the limb [2], and then inflated to a pressure capable of restricting venous return allowing arterial entry while exercising [3,4]. The physiological mechanisms that are activated by BFR are currently unclear. It has been hypothesized that an intramuscular hypoxic environment [5] could induce vascular endothelial growth and high levels of metabolic stress which could promote hypertrophy [6,7]. Furthermore, an increased concentration of growth factors, satellite cells, transcription factors [3], reactive oxygen species, intramuscular anabolic and anticatabolic reactions, and an increased recruitment of type II muscle fibers were also observed [6,8,9], which could facilitate muscle hypertrophy.

The quadriceps is the most affected muscle after injury and reconstruction of the anterior cruciate ligament (ACL) [10,11]; its rehabilitation reduces pain and improves knee function. In addition, its weakness is considered a main risk factor and an important indicator of the progression of knee osteoarthritis (KOA) [5,12], whereas an increase in strength of the quadriceps is related to less symptoms [13]. It has been stated that training with a high load increases strength and muscle mass in patients with atrophy [10]. The American College of Sports Medicine recommends loads of 60% to 70% of one maximum repetition (1MR) for strength gain and 70% to 85% of 1MR for hypertrophy [9,14,15]. However, this high load training has been found to cause pain and inflammation in ACL reconstruction and KOA patients [16,17], because the joint cannot withstand the amount of mechanical stress imposed during training.

The reviewed literature suggests that muscle hypertrophy occurs after training with BFR, in both healthy and injured people [18,19], achieving results similar to those of classic muscle mass gain training, but reducing joint stress and therefore increasing exercise tolerance [2,4]. Additionally, lower levels of pain and perceived exertion have been observed during and after BFR training. A recent systematic review [9] showed that BFR training is potentially effective in improving quadriceps strength in patients with weakness and atrophy related to knee pathology. Furthermore, the use of short duration, low-load resistance BFR training appears safe and not harmful after knee surgery or in KOA patients.

Given the increasing use of BFR training in KOA or ACL reconstruction patients, and the lack of qualitative synthesis of its effects, the aim of this systematic review is to examine the published literature on BFR training to analyze its effect on strength gains in ACL reconstruction and KOA patients. In addition, other secondary objectives are to analyze BFR’s effect on quadriceps cross-sectional area (CSA), pain intensity, and functionality and/or quality of life.

## 2. Materials and Methods

### 2.1. Protocol Design

To perform the present systematic review, we followed the recommendations in the Preferred Reporting Items for Systematic Reviews and Meta-Analyses (PRISMA) statement [20] and the Cochrane Handbook for Systematic Reviews of Interventions by Higgins et al. [21]. In addition, the methodological protocol of this review was previously registered in PROSPERO International Prospective Register of Systematic Review (id number: CRD42020182656).

### 2.2. Data Sources and Search Strategy

A systematic literature search was conducted by two authors (C.B.Á. and P.I.-K.S.), independently, in Web of Science (WoS), PEDro, Scopus, MEDLINE, Dialnet, CINAHL Complete and The Cochrane Library without limiting the date of publication. A further manual search of bibliographic references from the papers already extracted was done to identify potential studies not captured by the electronic database searches. Finally, grey literature was searched. Any discrepancies at this stage were resolved by a third author (A.A.-O.) with expertise in bibliographical searches. The entire search was conducted from April to June 2020. We searched (in the title and abstract fields) for a combination of terms (Table A1).

### 2.3. Study Selection and Eligibility Criteria

The search, compilation and selection of articles was carried out by two authors (C.B.Á. and P.I.-K.S.), who applied the same criteria to select the articles based on the population, intervention, comparison and outcomes (PICO). These two authors independently peer-reviewed the titles and abstracts of each article, then the same procedure was followed for the full-text review to identify the articles of interest for the present study. A third reviewer (A.A.-O.) resolved any discrepancies. Duplicate articles or in those in which it was not possible to obtain the full text were excluded from our study.

Articles that met the following inclusion criteria were included: the participants were men and/or women with ACL reconstruction or diagnosed with KOA with pai and/or some functional alteration; Randomized clinical trials (RCTs), which were in English or Spanish; The intervention consisted of BFR training, compared to training without BFR; Studies measured the results of at least one of the following parameters: muscle strength, quadriceps CSA, pain intensity or functionality and/or quality of life. Studies that were not RCTs, those in the process of publication that did not show full results and those conducted in children were excluded. To illustrate the selection process, we used the PRISMA flow chart [20].

### 2.4. Data Extraction

The outcomes considered in our study were strength, as the main variable, and the quadriceps CSA, pain intensity and functionality and/or quality of life as secondary variables.

Both reviewers (C.B.Á. and P.I.-K.S.) used the same form to extract the data from each study. The extracted data included the PICO elements: characteristics of the participants, sample size and study design, description of the interventions (rehabilitation protocol, type of BFR exercise and duration), as well as the outcome measures that were analyzed in each article and the results. Furthermore, data on the author, year and country of publication of the study were extracted. In the absence of data from one study, an attempt was made to contact the corresponding author(s) to obtain the incomplete information. If the corresponding author(s) could not be contacted, the article was excluded from the present study.

A meta-analysis was not possible due to the variability in the measurement of results, follow-up and characteristics of the studies included.

### 2.5. Study Quality and Risk of Bias

The methodological quality of the selected studies was assessed by both reviewers (C.B.Á. and P.I.-K.S.) independently using the PEDro scale [22,23]. To assess the risk of bias of the included articles we used the Cochrane risk of bias tool RoB-2 [24]. Disagreements were resolved by consulting a third reviewer (A.A.-O.). Categories included in the methodological assessment were: bias derived from the randomization process, bias due to deviations from planned interventions, bias due to lack of outcome data, bias in outcome measurement and selection bias in reported outcome, also including other possible sources of bias. Each item was classified based on the data described in the studies as low-risk of bias when the information coincided, unclear risk of bias when the information was not correct or was not complete or high risk of bias when the necessary information was not in the study.

## 3. Results

### 3.1. Included Studies

**Figure 1:**
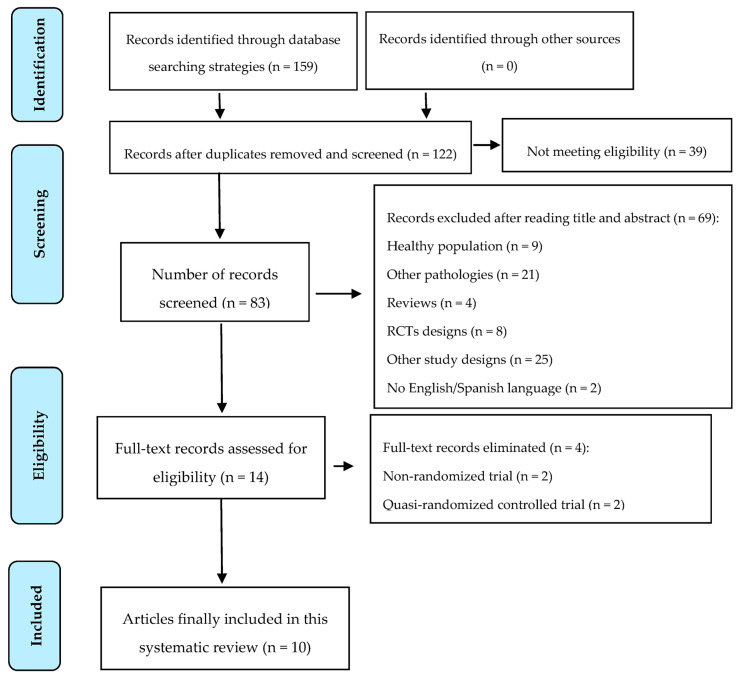
Preferred Reporting Items for Systematic Reviews and Meta-Analyses (PRISMA) flowchart diagram of the search process [20].

Figure 1 represents the flowchart of the study selection based on the PRISMA statement [20] (Figure 1). The initial search identified 159 studies and finally, 10 were included in this systematic review [25,26,27,28,29,30,31,32,33,34]. Finally, no study was suitable for conducting a meta-analysis.

### 3.2. Study Characteristics

All studies included in the review were RCTs [25,26,27,28,29,30,31,32,33,34] published from 2003 onwards. It should be noted that the studies from Hughes et al. [33] 2018 and Hughes et al. [34] 2019 are two reports from the same study. Two studies were conducted in Brazil [25,29], four in the USA [26,27,28,30], two in the UK [33,34], one in Norway [31] and one in Japan [32].

#### 3.2.1. Participants

Full descriptive details of the included studies are shown in Table 1. A total of 364 patients, 214 women and 146 men, participated in the 10 RCTs in the systematic review. In total, 166 patients received a BFR training intervention and 198 received a non-BFR training intervention. A population with two types of pathology were studied, where five studies included patients with KOA [25,26,27,28,29] (204 participants) and the other five studies included patients with ACL reconstruction [30,31,32,33,34] (160 participants). For the KOA population, the duration of the intervention was four weeks [27,28], six weeks [29] and 12 weeks [25]. In the ACL reconstruction population, the duration of intervention was two weeks [31], eight weeks [30,33,34] and 16 weeks [32].

#### 3.2.2. Intervention

The intervention in all studies consisted of applying BFR to the proximal thigh area while performing high-, medium- or low-load exercises. Low load was applied in nine studies that ranged from 20% [26] to 30% of 1MR [25,27,28,29,33,34]. One study applied low load but without specifying the percentage of MR used [32], another study only referred to the use of BFR together with an exercise protocol [31] and only one study applied BFR with a high load, at 70% of 1MR [31].

The interventions in the control groups consisted of performing the same exercises, but without BFR. Low-load training at 30% 1MR [27,28] or moderate load at 60% was used [26], although one study did not specify the percentage of low load [32]. Three studies applied high-load training at 70% of 1MR [29,30,34]. Furthermore, two studies presented two control groups [25,33]: one study [25] applied a load of 80% in one group and 30% in the other, and the other study [33] applied 70% of 1MR in one group and 30% in the other group in which BFR was also applied in healthy subjects. In addition, one of the studies did not specify the load [31].

#### 3.2.3. Comparison

Most of the studies compared the application of BFR with low load [25,26,27,28,29,32,33,34], high load [25,29,33,34], moderate load [26] or low load [25,27,28,32] non-BFR exercises. Only one study used high load in both the control and the intervention groups [30]. In addition, one study also compared the use of BFR in healthy subjects with low load [33], and another study did not specify the load but used the same exercise protocol with or without occlusion [31]. These data are shown in Table 1.

#### 3.2.4. Outcomes

The main outcome was muscle strength. Different methods were used for its evaluation, such as isokinetic force measurement [26,27,28,30,32], isotonic force [27,28] and maximum isometric contraction [29,30,32]. In addition, in two studies the 1MR leg press test was used [25,30].

As secondary data, the quadriceps CSA, pain and the functionality and/or quality of life of the patient were analyzed. Quadriceps CSA was examined in three studies, using computed tomography (CT) [25] or magnetic resonance imaging (MRI) [31,32]. To measure the intensity of pain in patients with KOA several scales were used, among them the Western Ontario and McMaster Universities Osteoarthritis Index (WOMAC) [25,26], the Knee Injury and Osteoarthritis Outcome Score (KOOS) [27,28] and the Numeric Pain Rating Scale (NPRS) [29]. To measure pain in patients with ACL reconstruction, the Borg scale was used to measure perceived muscle pain [33,34]. Different scales were used to evaluate the quality of life and functionality of the patient. In one study the International Knee Documentation Committee (IKDC) scale [30] was used; in others the WOMAC scale, the Short Form-36 health questionnaire (SF-36) [25] and the Lequesne questionnaire [29] were also used, in addition to the Late Life Function and Disability Instrument (LLFDI), Short Physical Performance Battery (SPPB) and change in gait speed [26]. In two studies the timed-stands test (TST) [25] and Timed Up and Go (TUG) [25,29] functionality tests were applied.

### 3.3. Study Quality and Risk of Bias

**Figure 2:**
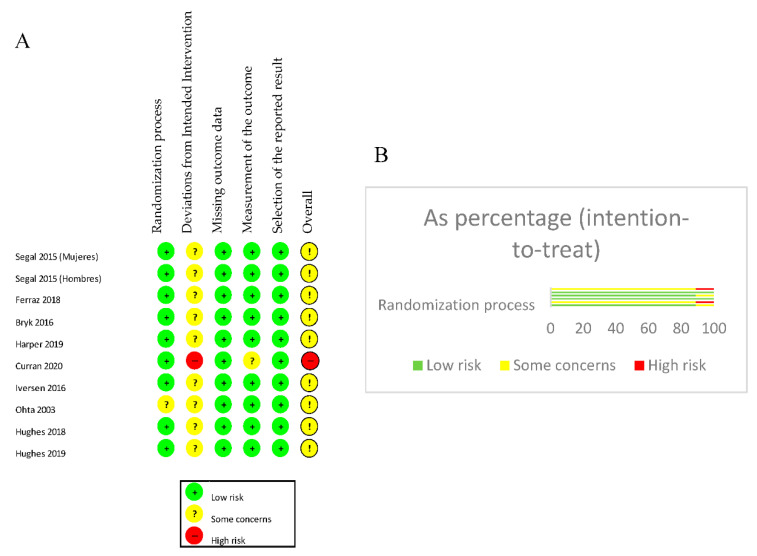
(**A**): Assessment of risk of bias. (**B**): Summary risk of bias by percentage.

The Cochrane “RoB-2” risk of bias tool [24] was used to assess the risk of bias in the included RCTs. When analyzing the domains of each study individually, 88.9% presented a low risk of bias in the randomization process, while 11.1% presented an unclear risk. An “unclear” risk of bias of the deviations from the planned interventions were observed in 88.9% and the measurement of the outcome presented a low risk of bias in 88.9%. Meanwhile the bias regarding the lack of outcome data and the selection of the reported outcome presented a low risk of bias in 100% of the studies. Regarding other sources of bias, 88.9% showed an “unclear” risk of bias, and 11.1% showed a high risk of bias. These results are shown in Figure 2.

Secondly, each RCT was analyzed based on the PEDro scale [22,23]; a score equal to or greater than six was obtained in nine of the studies, which represents good methodological quality. Only one study’s methodology was classified as poor quality since its score was less than four points (Table A2).

### 3.4. Outcomes

#### 3.4.1. Main Outcome: Muscle Strength

Ferraz et al. [25] evaluated the strength in women through the changes of 1MR of leg press and knee extension. Two control groups performed training with high or low load, and the intervention group performed training with low load, and BFR was studied. After 12 weeks, a significant increase in strength was observed in the BFR training group and in the high-load control group, compared to the low-load control group that did not show a significant increase in strength.

Segal et al. [27,28] studied the isotonic force in 1MR of the leg press and the maximal extensor isokinetic force after four weeks of training. In a study carried out in women [28] the 1MR isotonic force increased significantly in the BFR group compared to the control group (28.3 ± 4.8 kg and 15.6 ± 4.5 kg, respectively, *p* = 0.0385), and the isokinetic force also improved in the intervention group compared to the control group (0.07 ± 0.03 Nm/kg, −0.05 ± 0.03 Nm/kg respectively, *p* = 0.0048). In the study conducted in men [27], even though strength improved in both groups in the two measurements performed, there were no significant differences between the groups.

Bryk et al. [29] evaluated the strength in women through the maximum isometric contraction of the quadriceps. After six weeks of training, strength improved in the BFR group more than in the control group (40 ± 9.2 kg and 33.5 ± 12.9 kg, respectively; *p* = 0.001), but this difference was not significant between the groups. Harper et al. [26] studied the extensor isokinetic force. After 12 weeks of treatment, the force torque increased in the control group compared to the group with BFR 1.87 (−10.96, 7.23) Nm.

Ohta et al. [32] evaluated the changes in muscle strength of the hamstrings and quadriceps 16 weeks after ACL reconstruction. They did not differentiate between men and women while studying isokinetic force at 60°/s and 180°/s, and isometric force at 60°/s. The evaluation of flexor and extensor torques showed a significant increase in muscle strength in the BFR group compared to the control group (*p* < 0.05). The isokinetic strength of the extensors was greater in the BFR group compared to the control group, both at 60°/s (76 ± 16%, 55 ± 17%, respectively, *p* < 0.001) and at 180°/s (77 ± 13% and 65 ± 13%, respectively; *p* = 0.004). The strength in the knee flexors also showed better results in the BFR group than in the control group at 60°/s (81 ± 14% and 72 ± 15%, respectively; *p* = 0.05) and at 180°/s (84 ± 18% and 74 ± 12% respectively; *p* = 0.04). In addition, the evaluation of isometric strength at 60°/s also showed better results in the intervention group compared to the control group for extensor (84 ± 19% and 63 ± 19%, respectively; *p* < 0.001) and flexor (72 ± 11% and 62 ± 14%, respectively; *p* = 0.02) strength.

Finally, Curran et al. [30] evaluated isokinetic extensor muscle strength, isometric strength and change in 1MR leg press after ACL reconstruction. Both groups consisted of men and women who performed high-load training, including concentric and eccentric exercises. After eight weeks of treatment, there were no significant differences from before the operation to after the intervention between the BFR group and the control group or in the maximum isokinetic force, nor in isometric force (*p* = 0.49 and *p* = 0.88, respectively). There were also no significant differences between the groups in 1MR leg press from before the intervention to after (*p* = 0.24). These results are shown in Table 2.

#### 3.4.2. Secondary Outcomes: CSA, Pain, Function and/or Quality of Life

*● CSA*

There were three studies that measured the quadriceps CSA (results are shown in Table 3). The study of Ferraz et al. [25] showed significant increases in the BFR group (+7%, effect size (ES) = 0.39, *p* < 0.0001) and in the high-load control group (+8%, ES = 0.54, *p* < 0.0001), but there was no improvement in the low-load control group (+2%, ES = 0.12, *p* = 0.52). It is important to note that the increase in CSA between groups was significant compared to the low-load group (*p* = 0.02), but no significant difference was observed between the high load and the BFR groups (*p* > 0.05), showing a comparable effect between treatments for this variable. The study of Iversen et al. [31] observed that both groups (control group and BFR group) had a significant reduction in quadriceps CSA from two days before surgery to 16 days after surgery (*p* > 0.0001). Two measurements were taken and in both cases a reduction of the quadriceps CSA was observed both in the control group and in the BFR. During the intervention period (mean changes of 40% and 50%) the occlusion group lost 13.8% ± 1.1% (mean ± SEM) and the control group lost 13.1% ± 1.0% of the quadriceps CSA, respectively. There were no significant differences between the occlusion and control groups regarding quadriceps atrophy (*p* = 0.6205). The study by Ohta et al. [32] concluded that 16 weeks after a ACL surgery, there was a statistically significant increase in the CSA of the knee extensor muscles in the BFR group compared with the CG group (*p* < 0.05).

*● Pain*

Ferraz et al. [25] showed that the pain score according to the WOMAC subscale was significantly reduced in the low load and BFR groups (−45%, ES = −0.79, *p* = 0.001 and −39%, ES = −0.79, *p* = 0.02, respectively), but not in the high load group (−31%, ES = −0.54, *p* = 0.19). In the studies by Segal et al. [27,28] the KOOS scale was used to mediate pain. The study in women [28] observed that even though there was no worsening of knee pain in either group, there were no statistically significant differences between or within the groups (CG: 1.8 ± 2.7, *p* = 0.5209 and BFR: 2.0 ± 2.8, *p* = 0.4834) nor between the groups (*p* = 0.9574). In the study conducted with men [27], despite an improvement in pain in the control group and in the BFR (14.2 ± 7.2%, *p* = 0.062 and 4.9 ± 3.3%, *p* = 0.155, respectively), there was no significant difference between the two groups (*p* = 0.254). Bryk et al. [29] evaluated pain intensity with the NPRS scale. Although there was a slight improvement in the BFR group compared to the CG (3.2 ± 1.9, 3.5 ± 2.3 points, respectively; *p* = 0.001), there was no significant differences between the groups. Pain intensity during exercise was also evaluated, where pain significantly decreased within the groups, in the BFR group (2.5 ± 1.5 points) and the control group (6.2 ± 2.2 points), and improved between the groups (*p* = 0.01). The study conducted by Harper et al. [26] used the WOMAC pain sub-scale. They observed that the difference in both groups was −81 (−2.04, 0.42) points while between the groups there was a decrease of 0.24 (−2.51, 2.98) points in the control group compared to the BFR group. In the study conducted by Hughes et al. 2018 [33], muscle pain and knee pain were studied during the training session and 24 h after the training session. Muscle pain was significantly greater in the BFR group compared to the ACL reconstruction heavy-load group (mean difference: 5 ± 1, 95% confidence interval (CI): 2.942 to 7.758, *p* < 0.05) and compared to the group with healthy BFR individuals, (difference mean: 2.7 ± 1, 95% CI: 0.292–5.058, *p* < 0.05). However, knee pain improved in the BFR group as it was significantly less in the intervention group compared to the ACL reconstruction heavy-load group (difference mean 3 ± 1, 95% CI: 0.242–5.058, *p* < 0.01). In 2019, Hughes et al. [34] conducted another study with two groups of patients (participants selected from the previous study). They also studied the intensity of pain and knee pain during the training session and at 24 h. As in the first study, muscle pain increased significantly in the group that performed training with BFR (*p <* 0.05). In the last training session performed on the control group (*p <* 0.05, d = 0.8, 95% CI: 0.7–0.9) and the BFR resistance training group (*p <* 0.01, d = 0.5, 95% CI: 0.4–0.6), knee pain improved significantly in the BFR group both during the training session (*p <* 0.05) and 24 h after the training session (*p <* 0.05). The mean knee pain per session was lower in the BFR group compared to the heavy-load resistance training group during each session (all *p <* 0.05, mean d = 2.5, 95% CI: 2.2–2.8). In the BFR resistance training group knee pain in the middle session remained significantly lower compared to the beginning from the fourth session: (all *p <* 0.05, mean d = 1.2, 95% CI: 0.7–1.4). For the heavy-load resistance training group, mean knee pain remained significantly lower compared to the beginning from the sixth session (all *p <* 0.05, mean d = 0.6, 95% CI: 0.4–0.7). Pain at 24 h of training was less in the BFR resistance training group compared to the heavy-load resistance training group at all time points (all *p <* 0.01, mean d = 3.1, 95% CI: 2.9–3.3). In the BFR group, pain remained significantly lower from the fourth session (all *p <* 0.01, mean d = 2.9, 95% CI: 2.7–3.2) and in the control group, pain decreased significantly after from session 4 (all *p <* 0.01, mean d = 1.7, 95% CI: 1.3–2).

These results are shown in Table 4.

*● Function and/or quality of life*

Most of the studies analyzed showed improvements within the BFR group and control group, but in most cases, this improvement was not significant between the groups (results are shown in Table 5). The study conducted by Ferraz et al. [25] reported similar results in the BFR group and the high-load control group compared to the low-load control group. In the TST, both the BFR group and the control group with high load showed significant improvements after the intervention (7% and 14%, respectively) compared to the control group with low load (5%). Despite these effects within the group, no significant differences were observed between the three groups (*p* > 0.05), just as TUG had no difference either within groups or between groups (*p* > 0.05). The quality of life, evaluated by means of the total score of the WOMAC scale, showed a significant improvement in the three groups (*p* < 0.05). Bryk et al. [29] evaluated the functionality using the Lesquesne scale and the TUG. At the end of the treatment, the two groups improved in functionality (*p* = 0.001 and *p* = 0.006, respectively); although the score in the BFR group was better, this difference was not significant between groups. The study conducted by Harper et al. [26] showed similar results between the groups evaluated by SPPB, LLFDI and change in gait speed. The results obtained in the study conducted by Curran et al. [30] using the IKDC scale did not show significant improvements between groups (*p* > 0.05).

## 4. Discussion

The aim of this systematic review was to investigate whether people with KOA or ACL reconstruction who perform occlusive training have a greater increase in muscle strength and quadriceps CSA, and greater improvements in pain intensity and in functionality and/or quality of life, compared to those who undergo treatment without BFR. After conducting a thorough search, ten studies were included in our review, all of them RCTs.

Our main outcome was muscle strength. In recent years, several studies have shown increased muscle strength with the use of BFR in healthy subjects [35,36,37]. Although the physiological mechanisms of occlusive training remains unclear, it has been speculated that the hypoxic environment in the muscle leads to a series of changes at neuromuscular, hemodynamic, endocrine and metabolic levels that could lead to muscle hypertrophy [2]. Muscle weakness and atrophy is a common problem in patients with KOA and ACL surgery. People with KOA have been observed to experience pain and progressive functional loss [38] in activities of daily living [38,39]. There is consistent evidence that lower muscle quality, physical inactivity, joint degeneration and pain are associated with lower muscle strength in patients with KOA [40]. Furthermore, it has been shown that active exercise increases physical function and reduces pain and disability of the knee, improving health and the quality of life of the patient [39]. On the other hand, the main goals of surgery and rehabilitation in patients with ACL reconstruction are to restore knee function to pre-injury levels and promote long-term joint health. However, in many cases quadriceps weakness persists for months or even years after the operation [9]. Therefore, the rehabilitation of the quadriceps is essential to achieve good knee stability and maintain functional capacity [41]. Therefore, in both pathologies, to seek a strategy capable of minimizing quadriceps weakness is of great clinical interest. In our review, four of seven studies that assessed this variable showed improvements in strength [25,28,29,32]. In one study [29], this improvement was not significant compared to the control group. Ferraz et al. [25] showed similar improvement results compared to the high-load control group, but significantly improved compared to the low-load control group. Segal et al. [28] and Ohta et al. [32] also used low loads in both groups. Only the study by Bryk et al. [29] used loads of 70% of 1MR as a control group. Among the studies that did not show improvement, two of them presented as a control group loads between 60% [26] and 70% of 1MR [30]. In addition, Segal et al. [27] did not obtain significant differences after four weeks, despite applying a load of 30% of 1MR in men. It should be noted that, in all cases in which the increase in strength was significant, both the control and the intervention groups worked with low loads; in most of the studies where there were no differences, the control group used moderate or high loads.

The quadriceps CSA was evaluated in three studies [25,31,32]. Two of them showed significant increases in CSA [25,32]. In the study conducted by Ferraz et al. [25] the group that performed occlusion training improved compared to the low-load control group after 12 weeks. In the study conducted by Ohta et al. [32] the preoperative/postoperative relationship 16 weeks after surgery also showed an increase in knee extensor musculature. In contrast, Iversen et al. [31] observed a significant reduction of the quadriceps in athletes after undergoing ACL surgery; this may be because the measurement was made only 16 days after the operation.

Knee pain improved in four of seven studies that investigated this variable [25,29,33,34]. Each study used different scales. In the studies evaluating patients with KOA, there was an improvement in knee pain during exercise with BFR and at the end of treatment compared to training with high loads. In the study by Ferraz et al. [25] the pain score improved significantly in the BFR group and in the low-load control group; however, in the high-load control group 25% of the sample dropped out of the study due to pain related to exercise. The study conducted by Bryk et al. [29] showed improvements in pain intensity for both groups but obtained better results in the BFR group compared to the high-load control group. These data could indicate that training with low load and low pain could facilitate the adherence to exercise in patients with KOA. The studies conducted by Hughes et al. 2018 [33] and Hughes et al. 2019 [34] evaluated knee pain after ACL reconstruction during and after exercise. Both studies showed an improvement in the low-load BFR group compared to exercising with high loads. The results of Hughes et al. 2018 [33] cannot be generalized since only one session was conducted. Nevertheless posteriorly, Hughes et al. 2019 [34] performed a workout twice a week for eight weeks, showing significant improvement in knee pain. This indicates that exercise with low loads and the application of BFR can be a suitable alternative during the first phases of rehabilitation after an ACL reconstruction. It is important to note that in the rest of the studies, despite the lack of improvement compared to the control group, BFR training was not associated with worsening of knee pain in any of the cases [26,27,28].

The studies which evaluated the functionality and/or quality of life of the patient used different scales and questionnaires. One of four studies in which it was evaluated [25], obtained significant improvements in the BFR group after training. The assessment of quality of life using the WOMAC scale showed an improvement at the end of treatment in the three groups evaluated and the results of the functionality obtained an improvement in the BFR group and in the control group with high load. In the rest of the studies analyzed, despite not having significant improvements, there was no worsening of these parameters in the occlusive group [26,29,30].

The studies’ protocols showed high heterogeneity, regarding the cuff pressure, the series and repetitions performed, the time of application of BFR or the duration of the training. This heterogeneity could be observed in the high variability of cuff pressure between the studies analyzed, from a mean of 97.4 mmHg [25] to a maximum of 200 mmHg [27,28,29]. In some cases, the pressure gradually increased throughout treatment [27,28,31]. In addition, to determine the cuff pressure, some studies calculated it using 70% [25] and 80% [30,34] of total occlusion. Finally, one study individualized it to each patient according to the previous literature on BFR [26] and the others used pneumatic or manual devices. In addition, there were also variations in the width of the cuff used, the method for calculating arterial occlusive pressure (AOP) and the posture when applying the occlusion. These aspects are important since studies have concluded that depending on the width of the cuff, arterial flow is occluded at very different inflation pressures [42]. In the reviewed studies, the width cuff was either 6.5 cm [27,28], 11.5 cm [33,34], 14 cm [31] or 17.5 cm [25]; however, not all the studies indicated the width of the cuff used [26,29,30,32]. The method for calculating AOP was also different according to each study. Two studies used the Doppler probe to locate the tibial and femoral artery [25,29]. Recent hypotheses have indicated that cuff restrictive pressures in the lower body should be based on thigh circumference and not on pressures previously used in the literature [39,40], while other studies indicate that the pneumatic tourniquet system can be used to reliably calculate the AOP of the lower limb and adapt to muscular and vascular changes with results equal to the use of Doppler [43,44], considered the gold standard test. Finally, two studies in this review used the Doppler [33,34]. An important aspect that must be considered to calculate the correct AOP is the position of the body, since it must be measured in the position in which the occlusion stimulus will be applied later [44]. However only two of the reviewed studies applied it in this position [33,34], two others applied it in the supine position [25,30], two with the patients seated [28,31] and four of them did not indicate in which posture it was applied [26,27,29,32]. The optimal time of application of BFR is still not clear, which makes it difficult to standardize it when exercising. In five of the reviewed studies the pressure was maintained throughout the session, both in periods of exercise and in rest intervals [25,27,28,33,34]. In four of the studies the device remained inflated during exercise and deflated during rest periods [26,29,30,31], and in Ohta et al. [32], pressure was maintained during training for a maximum of 15 min and then deflated for 15–20 min. In addition, the duration of the rehabilitation protocol of the reviewed studies varied greatly with a minimum duration of one day [33] and a maximum of 16 weeks [32]. Finally, there was also great variability in the periodicity of the sessions from a single session per week [33], twice per week [25,30,34], three times per week [26,27,28,29], twice a day [31] or six sessions per week [32].

Adaptations of muscle strength and hypertrophy have been observed for periods of time greater than three weeks in duration [1]. According to the trials included in our study, a minimum of four weeks would be necessary to gain muscle strength or CSA. Nevertheless, more studies are needed on the duration and frequency of training, since these parameters are highly variable between the studies. These aspects should be considered for future studies, and a standardized protocol can be established.

This systematic review has several limitations. There is a high clinical and statistical heterogeneity between the included studies. The differences in the time of application of the BFR, intervention protocols and way of measuring the variables has made it impossible to conduct a meta-analysis. In some studies, the sample size to be studied was small, and one of the included studies was a pilot RCT [26], which may lead to the risk of over-interpreting the results obtained. Despite having carried out searches in several databases by the two authors independently, it is possible that an article that may be relevant was not included. Furthermore, our study shows an “unclear” risk of bias in 89% of the studies, in the deviations from the planned interventions and in other possible sources of bias, data that must also be considered when interpreting the results.

## 5. Conclusions

After performing a systematic review to assess the effects of BFR training compared to non-BFR training on quadriceps strength, cross-sectional area (CSA), pain perception, function and quality of life on patients with KOA and under ACL reconstruction, the reviewed evidence could suggest that BFR training may benefit muscle strength and CSA similar to high-intensity training. Furthermore, it could also have similar results to low-intensity training in terms of quality of life and knee pain intensity during exercise. Taken together, it could suggest that BFR training could provide benefits similar to high-intensity training, without the detriment of this type of work, and with the advantages of low-intensity training. However, more research is needed to confirm these conclusions about BFR treatment and to establish a standardized intervention protocol for future trials.

## Figures and Tables

**Table 1:** Characteristics of the studies included in the review.

Author/Year	Study Design	Participants Characteristics	Intervention/Rehabilitation Protocol	Pressure (mmHg)	Measure Time Points	Outcome Measures, Units
Ferraz et al., 2018 [25]	RCT	- OA - Women - *n* = 48 - 3 groups: CG1: HI-RT (80% 1MR) MY: 59.9 ± 4 *n* = 16 CG2: LI-RT (30% 1MR) MY: 60.7 ± 4 *n* = 16 IG: LI-RT + BFR (30% 1MR + BFR) MY: 60.3 ± 4 *n* = 16	12 weeks, 2 times per week Bilateral leg press and knee extension exercises - 1st week: HI-RT = 4 × 10 (50%) LI-RT = 4 × 15 (20% 1MR) LI-RT + BFR = 4 × 15 (20% 1MR) - 2nd week: HI = 4 × 10 (80% 1MR) LI = 4 × 15 (30% 1MR) LI-RT + BFR = 4 × 15 (30% 1MR) - 5th week: HI = 5 × 10 (80% 1MR) LI = 5 × 15 (30% 1MR) LI-RT + BFR = 5 × 15 (30% 1MR) 1 rest between sets BFR is kept through exercise and rest	70% BFR Media: 97.4 ± 7.6 mmHg	12 weeks	Strength: - 1MR leg press - 1MR knee extension CSA quadriceps: - CT Function: - TST (s) - TUG (s) - Sub scale WOMAC functional capacity (17 items, 0–68 points) Quality of life: - WOMAC (0–100 points) - SF-36 (0–100) Pain: - Sub scale WOMAC pain (5 items, 0–20 points)
Harper et al., 2019 [26]	RCT pilot	- OA - Men and women - *n* = 35 - 2 groups: CG: MIRT (60% 1MR) *n* = 19 (15 women and 4 men) MY: 69.1 ± 7.1 IG: BFR (20% 1MR) *n* = 16 (10 women and 6 men) MY: 67.2 ± 5.2	12 weeks, 3 times per week 4 exercises: leg press, leg extension, calf flexion and leg flexion. Repetitions were carried out until muscular fatigue. CG: 60% 1MR IG: 20% 1MR with BFR BFR is kept through exercise and deflates in the rest periods	Individualized to each patient	12 weeks	Strength: - isokinetic extensor strength (Nm) Function: - SPPB (0–12 points) - walking speed (m/s) Disability and function: - LLFDI (0-100 points) Pain: - Sub scale of pain WOMAC (0–20)
Segal et al., 2015 [28]	RCT	- OA - Women - *n* = 45 - 2 Groups: CG: low load (30% 1MR) *n* = 24 MY: 54.6 ± 6.9 IG: BFR + low load *n* = 21 MY: 56.1 ± 5.9	4 weeks, 3 times per week 4 set of bilateral leg press (1× 30 and 3× 15 reps with 30 s rest between sets) CG: 30% 1MR IG: 30% 1MR with BFR Pressure is maintained during exercise and rest (5 min of occlusion during exercise and 1.5 min of rest)	160–200 mmHg	4 weeks	Strength - isokinetic bilateral press strength (kg/kg) - max. isokinetic extensor strength (Nm/kg) Pain: - KOOS scale (0–100 points)
Segal et al., 2015 [27]	RCT	- OA - Men - *n* = 42 - 2 groups: CG: low load (30% 1MR) *n* = 22 MY: 56.1 ± 7.7 IG: BFR + low load *n* = 20 MY: 58.4 ± 8.7	4 weeks, 3 times per week 4 set of bilateral leg press (1× 30 and 3× 15 reps with 30 s rest between sets) CG: 30% 1MR IG: 30% 1MR with BFR Pressure is maintained during exercise and rest (5 min of occlusion during exercise and 1.5 min of rest)	160–200 mmHg	4 weeks	Strength: - isokinetic bilateral press strength (kg/kg) - max. isokinetic extensor strength (Nm/kg) Pain: - KOOS scale (0–100 points)
Bryk et al., 2016 [29]	RCT	- OA - Women - *n* = 34 - 2 Groups: CG: heavy load (70% 1MR) *n* = 17 MY: 60.4 ± 6.7 IG: BFR + low load (30% 1MR) *n* = 17 MY: 62.3 ± 7.0	6 weeks, 3 times per week Strengthening and stretching of LE CG: Quadriceps strengthening 70% 1MR IG: Quadriceps strengthening 30% 1MR with BFR Pressure is held only during exercise	200 mmHg	6 weeks	Strength: - MVIC Pain: - NPRS (0–10 cm), 2 measures: 1. Pain intensity 2. Pain during exercise. Function: - Lequesne questionnaire (0–20 points) - TUG (s)
Curran et al., 2020 [30]	RCT	- ACL reconstruction - Men (*n* = 15) and women (*n* = 19) - *n* = 34 4 Groups: CG1: concentric *n* = 8 (3 men and 5 women) MY: 6.1 ± 2.6 G2: eccentric *n* = 8 (2 men and 5 women) MY: 18.8 ± 3.9 IG1: BFR + concentric *n* = 9 (5 men and 4 women) MY: 15.3 ± 0.9 IG2: BFR + eccentric *n* = 9 (5 men and 4 women) MY: 16.0 ± 1.7 Later: 2 groups: CG: non-BFR *n* = 16 (5 men and 11 women) MY: 17.4 ± 3.5 IG: BFR *n* = 18 (10 men and 8 women) MY: 15.7 ± 1.3	8 weeks, 2 times per week 5 sets of 10 reps 2 min rest between sets. Isokinetic leg press concentric or eccentric 70% 1MR with or without BFR. Training starts 10 weeks after surgery. CG: Heavy load 70% 1MR, concentric or eccentric IG: Heavy load 70% 1MR concentric or eccentric with BFR Pressure is held only during exercise and released during rest and between sets 4 measurements: 1. Pre-operative (2 weeks before surgery) 2. Pre-intervention 3. Post-intervention (2 weeks after finishing the intervention) 4. Return to activity (RTA)	80% BFR (110–186 mmHg)	20 weeks	Strength: - Quadriceps MVIC - Quadriceps isokinetic strength - 1MR leg press Function: -IKDC scale (0–100 points)
Iversen et al., 2016 [31]	RCT blind	- ACL reconstruction (athletes) - *n* = 24 subjects - ACL injury with less than 6 months pre-surgery. - 2 groups: CG: exercise *n* = 12 (7 men and 5 women) MY: 29.8 ± 9.3 IG: BFR + exercise *n* = 12 (7 men and 5 women) MY: 25.9 ± 7.4	2 weeks 5 set of 20 reps quadriceps exercises with or without BFR. IG: bent trunk 45°, 5 reps, 2 times per day. Quadriceps exercise with occlusion (isometric contractions, progressing to knee extension and leg raise with knee straight. 20 reps every 5 min of occlusion. (100 reps per session, 200 per day). Intervention 2 weeks after surgery. CG: Same exercises without BFR Pressure is held during exercise (5 min) and released during rest (3 min)	130–180 mmHg (first day 130 mmHg. 10 mmHg increase every two days until reaching 180 mmHg)	16 days post-surgery	Quadriceps CSA: MRI (2 days before surgery and 16 days after surgery)
Ohta et al., 2003 [32]	RCT prospective	- ACL with semitendinosus auto graft - *n*: 44 patients (25 men and 19 women) -Age 18–52 years -2 groups: CG: exercise: *n* = 22 (12 men and 10 women) MY: 30 ± 9.7 IG: BFR + exercise *n* = 22 MY: 28 ± 9.7	16 weeks Week 1: both groups just exercise Week 2: IG exercise with BFR Exercise: leg raise with knee extension and hip abduction for 5 s 20 reps Exercises twice a day, 6 times per week for 8 weeks after the surgery (weeks 2–4 add 1 kg to the foot; weeks 5–8 add 2 kg Hip adduction exercise (hold ball between two knees) and squat exercise for 5 s 20 reps 2 times per day, 6 times per week. weeks 1–12 post-surgery, 16 weeks post ACL reconstruction. Exercises LE. 1–3 sets × day. 6 times per week. 20–60 reps. 14 weeks (first 2 weeks after surgery without BFR) Occlusion during training for a maximum of 15 min, then rest without inflation for 15–20 min	180 mmHg	16 weeks post-surgery	Measurements before and 16 weeks after surgery. Muscle strength: Isokinetic and isometric knee extensors and flexors: Dynamometer CSA of extensor and flexor muscles + adductor muscles: MRI
Hughes et al., 2018 [33]	RCT	- ACL with semitendinosus auto graft - *n* = 30 - Age: 23–39 years (23 men and 7 women) - 3 groups: CG1: No injured BFR + low-load exercise (30% 1MR) (NI-BFR). *n* = 10 (10 men) MY: 28 ± 5 CG2: ACL surgery with heavy-load exercises (70% 1MR). (ACLR-HL) *n* = 10 (7 men and 3 women) MY: 31 ± 7 IG: ACL surgery with BFRv+ low-load exercise (30% 1MR) (ACLR-BFR) *n* = 10 (6 men and 4 women) MY: 29 ± 5	Single session 5 min warm-up of pedal stroke followed by 10 reps of one-sided press with self-selected weight and 5 min of rest. ACL patients work on the operated side and patients without ACL work with the dominant limb. NI-BFR (CG1) and ACLR-BFR (IG) did 4 sets (30, 15, 15 and 15 reps, with 30 s rest between sets) unilateral leg press 30% 1MR from 0° to 90° ROM with 1 s concentric/1 s eccentric with BFR 80%. - ACLR-HL group unilateral leg press (3× 10 reps with 30 s rest between sets) form 0°–90° ROM 70% 1MR BFR during exercise and rest	NI-BFR: 173 mmHG ACLR-BFR: 186 mmHG	23 days post-surgery	Pain: - Muscular (0–11 points) - Knee: pain scales Muscle pain was evaluated in all 3 groups. Knee pain was evaluated in ACLR-BFR and ACLR-HL groups
Hughes et al., 2019 [34]	RCT	- ACL surgery - *n*: 28 Selected in a previous study (Hughes et al. 2018) - 2 groups: CG: HL-RT heavy load 70% 1MR *n* = 14 (10 men and 2 women) MY: 29 ± 7 IG: BFR-RT low load (30% 1MR) *n* = 14 (7 men and 5 women) MY: 29 ± 7	8 weeks, 16 sessions, 2 times per week Evaluation to start 48 h after the surgery. Unilateral leg press. Familiarization session with 5 min warm-up with unloaded bike. CG: 3× 10 reps. 30 s rest between sets, unilateral leg press, from 0°–90°, 70% 1M IG: 4 sets (30, 15, 15 and 15 reps) unilateral leg press Both limbs were trained. Occlusion was applied during exercise and rest time.	- 80% BFR - 150 mmHg operated lower limb - 157 mmHg healthy lower limb	8 weeks	Pain: - Muscular: Borg scale (last session measurement) - Knee: pain scales. 24 h after the session and during the session. Measurement of the average of the last sessions.

Abbreviations: RCT, randomized controlled trial; OA, osteoarthritis; ACL, anterior cruciate ligament; ACLR, anterior cruciate ligament reconstruction; BFR, blood flow restriction; 1MR, 1 maximum repetition; MY, mean years; CG, control group; IG, intervention group; HI-RT, high-intensity resistance training; LI-RT, low-intensity resistance training; MRI, magnetic resonance imaging; CT, computed tomography; WOMAC, Western Ontario and McMaster Universities Osteoarthritis Index; TST, timed-stands test; TUG, Timed Up and Go; CSA, quadriceps cross-sectional area; SF-36, Short Form-36 health questionnaire; MIRT, moderate-intensity resistance training; SPPB, Short Physical Performance Battery; LLFDI, Late Life Functional And Disability Instrument; KOOS, Knee Injury and Osteoarthritis Outcome Score; NPRS, Numeric Pain Rating Scale; MVIC, maximum voluntary isometric contraction; IKDC, International Knee Documentation Committee; SEBT, star excursion balance test; LE, lower extremity; BFR-RE, blood flow restriction resistance exercise; NI-BFR, non-injured BFR-RE; ACLR-BFR, ACLR patients’ blood flow restriction resistance exercise; ACLR-HL, ACLR patients heavy-load resistance exercise; ROM, range of motion; BFR-RT, blow flow restriction resistance training; HL-RT, heavy-load resistance training.

**Table 2:** Strength outcomes.

Author	Measurement	Intra-Groups	Between-Groups
Ferraz et al. [25]	1MR leg press (% improvement)		
	BFR:	26%; ES = 1.01	BFR vs. LI -RT: 18% BFR vs. HI-RT: −7%
	HI-RT:	33%; ES = 0.82	HI-RT vs. LI-RT: 21%
	LI-RT:	8%; ES = 0.23	
	1MR knee extension (% improvement)		
	BFR:	23%; ES = 0.86	BFR vs. LI -RT: 16% BFR vs. HI-RT: 1%
	HI-RT:	22%; ES = 0.83	HI-RT vs. LI-RT: 15%
	LI-RT: 7%	7%; ES = 0.21	
Segal et al. Women [28]	Extensor isokinetic strength (Nm/kg) pre-post treatment changes		
	BFR:	0.07 ± 0.03	0.57 ± 0.6
	CG:	−0.5 ± 0.3	
	Isotonic. 1MR leg press kg. pre-post treatment changes		
	BFR:	0.4 ± 0.3	0.2 ± 0.6
	CG:	0.2 ± 0.3	
Segal et al. Men [27]	Extensor isokinetic (% improvement Nm/kg)		
	BFR:	−0.1 ± 3.3 (0.4 ± 2.4%)	−0.8 ± 0.3 (−6.3 ± 0.1%)
	CG:	7.0 ± 3.0 (6.7 ± 2.3%)	
	Isotonic. 1MR leg press (% improvement kg)		
	BFR:	11.3 ± 14.0 (3.1 ± 0.9%)	−1.7 ± −2.8 (1.6 ± −0.4)
	CG:	13 ± 16.8 (4.7 ± 1.3%)	
Curran et al. [30]	Extensor isokinetic. Nm (pre-surgery to post-intervention changes)		
	BFR:	−12.4 ± 19.2	2.6 ± −15.2
	CG:	−15.0 ± 34.4	
	MVIC. Nm (pre-surgery to post-intervention changes)		
	BFR:	−16.3 ± 3.1	4.5 ± −35.2
	CG:	−11.8 ± 38.3	
	1MR leg press. kg (pre-intervention a post-intervention)		
	BFR:	2.44 ± 1.21	0.34 ± 0.26
	CG:	2.10 ± 0.95	
Ohta et al. [32]	Extensors (concentric 60° and 180°, isometric at 180°) (% post)		
	BFR:	C60° = 76 ± 16%, C180° = 77 ± 13% IS 60° = 84 ± 19	C60°: 21 ± −1% C180°: 12 ± 0% IS60°: 21 ± 0%
	CG:	C60° = 55 ± 17%, C180° = 65 ± 13% IS 60° = 63 ± 19%	
Bryk et al. [29]	MVIC. kg Post		
	BFR:	16.8 ± 10.3 (10.6, 22.9)	7.4 (0.9, 13.9)
	CG:	9.4 ± 8.3 (1.3, 17.5)	
Harper et al. [26]	Isokinetic. Nm (mean change) 95% CI		
			6-weeks: 9.96 (5.76, 14.16) 12-weeks: −1.87 (−10.96, 7.23)

Abbreviations: BFR, blood flow restriction; 1MR, 1 maximum repetition; CG, control group; kg, kilograms; Nm, Newton meter; HI-RT, high-intensity resistance training; LI-RT, low-intensity resistance training; MVIC, maximum voluntary isometric contraction; ES, effect sizes; C, concentric; IS, isometric.

**Table 3:** Quadriceps CSA outcomes.

Author	Measurement	Intra-Groups	Between-Groups
Ferraz et al. [25]	CT (% improvement)		
	BFR:	7%; ES = 0.39	BFR vs. LI -RT: 5% BFR vs. HI-RT: −1%
	HI-RT:	8%; ES = 0.54	HI-RT vs. LI-RT: 6%
	LI-RT:	2%; ES = 0.12	
Ohta et al. [32]	% Post		
	BFR:	101 ± 11%	9 ± (−1)
	CG:	92 ± 12%	
Iversen et al. [31]	Mean change (%)		
	BFR:	−13.8 ± 1.1%	0.7 ± 0.1
	CG:	−13.1 ± 1.0%	

Abbreviations: BFR, blood flow restriction; CG, control group; HI-RT, high-intensity resistance training; LI-RT, low-intensity resistance training; ES, effect sizes; Sig., significant; CT, computed tomography.

**Table 4:** Function and/or quality of life outcomes.

Author	Measurement	Intra-Groups	Between-Groups
Ferraz et al. [25]	TST (% improvement)		
	BFR:	7%; ES = 0.43	BFR vs. LI-RT: 2% BFR vs. HI-RT: −7%
	HI-RT:	14%; ES = 0.52	HI-RT vs. LI-RT: 9%
	LI-RT:	5%; ES = 0.32	
	WOMAC (quality of life) total score. Post intervention		
	BFR:	17.1 ± 11.2 (−46%); ES = −1.3	BFR vs. LI-RT: −1.3 ± −0.3 (−4%) BFR vs. HI-RT: −4.1 ± 2.0 (−7%)
	HI-RT:	21.2 ± 13.2(−39%); ES = −1.23	HI-RT vs. LI-RT: 2.8 ± 1.7 (3%)
	LI-RT:	18.4 ± 11.5 (−42%); ES = −0.79	
Curran et al. [30]	IKDC		
	Change from pre-operation to post-intervention:		−6.06 ± 0.53
	BFR:	11.83 ± 16.67	
	CG:	17.89 ± 17.20	
	Change from pre-intervention to post-intervention		
	BFR:	16.49 ± 9.97	−3.99 ± −2.3
	CG:	20.48 ± 12.27	
Bryk et al. [29]	Lequesne questionnaires. Post intervention		
	BFR:	−5.0 ± 4.5 (−2.8, 7.2)	1.0 (−3.3, 5.3)
	CG:	−6.0 ± 7.5 (−1.5, 10.5)	
	TUG (s) POST		
	BFR:	−1.2 ± 1.8 (−2.9, −0.21)	0.4 (−1.5, 2.3)
	CG:	−1.6 ± 3.5 (0, −3.2)	
Harper et al. [26]	SPPB (mean change in points)		
		(BFR decreased compared to CG)	−0.66 (−1.74, 0.42)
	Walking speed (m/s) mean change		
		(BFR decreased compared to CG)	−0.01 (−0.11, 0.09) m/s
	LLFDI. Mean change		
		(BFR decreased compared to CG)	−0.79 (−6.76, 5.17)

Abbreviations: BFR, blood flow restriction; CG, control group; HI-RT, high-intensity resistance training; LI-RT, low-intensity resistance training; WOMAC, Western Ontario and McMaster Universities Osteoarthritis Index; TST, timed-stands test; TUG, Timed Up and Go; SPPB, Short Physical Performance Battery; LLFDI, Late Life Functional and Disability Instrument; IKDC, International Knee Documentation Committee; ES, effect sizes; Sig., significant; m/s, meters/seconds.

**Table 5:** Pain outcomes.

Author	Measurement	Intra-Groups	Between-Groups
Ferraz et al. [25]	WOMAC pain sub scale. POST (% decrease)		
	BFR:	4.0 ± 2.9 (−39%); ES = −0.79	
	HI-RT:	4.6 ± 3.1 (−45%); ES = −0.59	
	LI-RT:	4.0 ± 2.6 (−31%); ES = −0.70	
Segal et al. Women [28]	KOOS (change pre-post)		
	BFR:	2.0 ± 2.8	0.2 ± 0.1
	CG:	1.8 ± 2.7	
Segal et al. Men [27]	KOOS (change pre-post)		
	BFR:	5.6 ± 11.7 (4.9 ± 3.3%)	2.7 ± 1.7 (−9.3 ± −3.9)
	CG:	2.9 ± 10.0 (14.2 ± 7.2%)	
Bryk et al. [29]	NPRS knee pain intensity Post		
	BFR:	−3.3 ± 2.2 (−4.8, −1.7)	−0.8 (2.2, 0.6)
	CG:	−2.5 ± 1.8 (−4.2, −0.8)	
	NPRS in exercise		
	BFR:	2.5 ± 1.5	−3.7 (−5.0, −2.4)
	CG:	6.2 ± 2.2	
Harper et al. [26]	WOMAC pain sub scale.		
		CG decreased compared to BFR.	0.24 (−2.51, 2.98)
Hughes et al., 2018 [33]	Muscular pain. Mean difference		
	a. BFR greater pain compared to healthy CG. b. BFR greater pain compared to heavy-load CG. c. Healthy greater pain compared to heavy-load CG.		a. 2.7 ± 1 (95% CI: 0.292–5.058) b. 5 ± 1 (95% CI: 2.942–7.758) c. 3 ± 1 (95% CI: 0.242–5.058)
	Knee pain during session and at 24 h		
	BFR lower pain compared to heavy-load CG.		3 ± 1 (95% CI: 0.242–5.058)
Hughes 2019 [34]	Muscular pain.		
	More pain with BFR		
	BFR: last session Injured limb	Mean d = 0.5 (95% CI: 0.4–0.6)	*p* < 0.05 *
	Non- Injured limb	Mean d = 1.0 (95% CI: 0.8–1.2)	
	CG: last session Injured limb	Mean d = 0.8 (95% CI: 0.7–0.9)	
	Non-Injured limb	Mean d = 0.7 (95% CI: 0.6–0.8)	
	Knee pain at 24 h. Sessions average		
	Less pain with BFR		Mean d = 3.1 (95% CI: 2.9–3.3)
	BFR injured limb: average from all sessions	Mean d = 2.9 (95% CI: 2.7–3.2)	
	CG injured limb: average from all sessions	Mean d = 1.7 (95% CI: 1.3–2.0).	
	Knee pain during session		
	Less pain with BFR		Mean d = 2.5 (95% CI: 2.2–2.8)
	BFR injured limb: average from all sessions	Mean d = 1.2 (95% CI: 0.7–1.4)	
	CG injured limb: average from all sessions	Mean d = 0.6 (95% CI: 0.4–0.7)	

Abbreviations: BFR, blood flow restriction; CG, control group; HI-RT, high-intensity resistance training; LI-RT, low-intensity resistance training; WOMAC, Western Ontario and McMaster Universities Osteoarthritis Index; KOOS, knee injury and osteoarthritis outcome Score; NPRS, numeric pain rating scale; ES, effect sizes; Sig., significant; CI, confidence interval; d, Cohen; *, numerical data not available.

## Appendix A

**Table A1:** Research strategy.

WoS	Knee AND (“blood flow occlusion training” OR kaatsu OR “blood flow restricted exercise”) Knee osteoarthritis AND (“blood flow restricted exercise” OR kaatsu) “Occlusion training” AND (“knee osteoarthritis” OR “Anterior Cruciate Ligament”) “Blood flow restricted exercise” AND (knee osteoarthritis OR ACL)
PEDro	“Blood flow restricted” AND knee Kaatsu AND knee “Knee osteoarthritis” AND BFR “Anterior cruciate ligament” AND BFR
Scopus	(“Blood flow occlusion” OR “blood flow restricted” OR kaatsu) AND “knee osteoarthritis” (“Blood flow occlusion” OR “blood flow restricted” OR kaatsu) AND ACL (“Blood flow restriction exercise” OR “occlusion training” OR “ischemic strength training”) AND knee
MEDLINE (Pubmed)	(“Blood flow restriction” OR “occlusion training” OR kaatsu) AND knee (“Blood flow restriction” OR “occlusion training” OR kaatsu) AND “Anterior Cruciate Ligament” “Blood flow restriction” AND ACL “Blood flow restriction” AND “knee osteoarthritis” “knee osteoarthritis” AND (“occlusion training” OR Kaatsu) BFR AND (“knee osteoarthritis” OR “anterior cruciate ligament”)
Dialnet	knee AND (“blood flow restriction exercise” OR kaatsu) (“Blood flow restriction” OR kaatsu OR “blood flow occlusion”) AND knee
Cochrane Library	“Blood flow restricted exercise” AND knee (“Blood flow restriction” OR “occlusion training” OR kaatsu) AND “knee osteoarthritis” (“Blood flow restriction” OR “occlusion training” OR kaatsu) AND ACL (“Blood flow restriction” OR “occlusion training” OR kaatsu) AND “anterior cruciate ligament” BFR AND “anterior cruciate ligament” BFR AND “knee osteoarthritis”
CINAHL (EBSCO)	Knee AND (“blood flow restriction exercise” OR kaatsu) (blood flow restricted” OR “blood flow occlusion” OR kaatsu) AND “knee osteoarthritis” (blood flow restricted” OR “blood flow occlusion” OR kaatsu) AND “anterior cruciate ligament”

**Table A2:** PEDro scale.

	Eligibility Criteria Specified	Random Allocation	Concealed Allocation	Groups Similar at Baseline	Subject Blinding	Therapist Blinding	Assessor Blinding	Less than 15% Dropouts	Intention-To-Treat Analysis	Between-Group Statistical Comparisons	Point Measures and Variability Data	TOTAL
Ferraz et al.	No	Yes	No	Yes	No	No	Yes	No	Yes	Yes	Yes	6
Harper et al.	Yes	Yes	No	Yes	No	No	Yes	No	Yes	Yes	Yes	7
Segal et al. (M)	Yes	Yes	Yes	Yes	No	No	Yes	Yes	No	Yes	Yes	8
Segal et al. (H)	Yes	Yes	Yes	Yes	No	No	Yes	Yes	No	Yes	Yes	8
Bryk et al.	No	Yes	Yes	Yes	No	No	Yes	No	No	Yes	Yes	6
Curran et al.	Yes	Yes	No	Yes	No	No	No	Yes	No	Yes	Yes	6
Iversen et al.	No	Yes	No	Yes	No	No	Yes	Yes	No	Yes	Yes	6
Ohta et al.	No	Yes	No	Yes	No	No	No	No	No	Yes	Yes	4
Hughes et al., 2018	Yes	Yes	Yes	Yes	No	No	Yes	Yes	No	Yes	Yes	8
Hughes et al., 2019	No	Yes	Yes	Yes	No	No	Yes	Yes	No	Yes	Yes	7

## References

[1] Patterson S.D., Hughes L., Warmington S., Burr J., Scott B.R., Owens J., Abe T., Nielsen J.L., Libardi C.A., Laurentino G. (2019). Blood flow restriction exercise position stand: Considerations of methodology, application, and safety. Front. Physiol..

[2] Flores-García L.A. (2019). El entrenamiento con oclusión vascular (EOV) como alternativa en rehabilitación muscular. Rev. Sanid. Milit..

[3] Loenneke J.P., Wilson J.M., Wilson G.J., Pujol T.J., Bemben M.G. (2011). Potential safety issues with blood flow restriction training. Scand. J. Med. Sci. Sports.

[4] Cuyul-Vásquez I., Leiva-Sepúlveda A., Catalán-Medalla O., Araya-Quintanilla F., Gutiérrez-Espinoza H. (2020). The addition of blood flow restriction to resistance exercise in individuals with knee pain: A systematic review and meta-analysis. Braz. J. Phys. Ther..

[5] Hughes L., Paton B., Rosenblatt B., Gissane C., Patterson S.D. (2017). Blood flow restriction training in clinical musculoskeletal rehabilitation: A systematic review and meta-analysis. Br. J. Sports Med..

[6] Pearson S.J., Hussain S.R. (2015). A Review on the Mechanisms of Blood-Flow Restriction Resistance Training-Induced Muscle Hypertrophy. Sport. Med..

[7] Singer T.J., Stavres J., Elmer S.J., Kilgas M.A., Pollock B.S., Kearney S.G., McDaniel J. (2020). Knee extension with blood flow restriction: Impact of cuff pressure on hemodynamics. Eur. J. Appl. Physiol..

[8] Pujol T.J., Loenneke J.P. (2009). The Use of Occlusion Training to Produce Muscle Hypertrophy. Strength Cond. J..

[9] Barber-Westin S., Noyes F.R. (2019). Blood Flow-Restricted Training for Lower Extremity Muscle Weakness due to Knee Pathology: A Systematic Review. Sport. Heal. A Multidiscip. Approach.

[10] Wilkinson B.G., Donnenwerth J.J., Peterson A.R. (2019). Use of Blood Flow Restriction Training for Postoperative Rehabilitation. Curr. Sports Med. Rep..

[11] Akima H., Furukawa T. (2005). Atrophy of thigh muscles after meniscal lesions and arthroscopic partial menisectomy. Knee Surgery Sport. Traumatol. Arthrosc..

[12] Petterson S.C., Barrance P., Buchanan T., Binder-Macleod S., Snyder-Mackler L. (2008). Mechanisms Underlying Quadriceps Weakness in Knee Osteoarthritis. Med. Sci. Sport. Exerc..

[13] Segal N.A., Torner J.C., Felson D., Niu J., Sharma L., Lewis C.E., Nevitt M. (2009). Effect of thigh strength on incident radiographic and symptomatic knee osteoarthritis in a longitudinal cohort. Arthritis Rheum..

[14] Communications S. (2009). Progression Models in Resistance Training for Healthy Adults. Med. Sci. Sport. Exerc..

[15] Garber C.E., Blissmer B., Deschenes M.R., Franklin B.A., Lamonte M.J., Lee I.-M., Nieman D.C., Swain D.P. (2011). Quantity and Quality of Exercise for Developing and Maintaining Cardiorespiratory, Musculoskeletal, and Neuromotor Fitness in Apparently Healthy Adults. Med. Sci. Sport. Exerc..

[16] Cerqueira M.S., de Brito Vieira W.H. (2019). Effects of blood flow restriction exercise with very low load and low volume in patients with knee osteoarthritis: Protocol for a randomized trial. Trials.

[17] Jan M., Lin J.-J., Liau J., Lin Y.-F., Lin D. (2008). Investigation of clinical effects of high- and low-resistance training for patients with knee osteoarthritis: A randomized controlled trial. Phys. Ther..

[18] Lu Y., Patel B.H., Kym C., Nwachukwu B.U., Beletksy A., Forsythe B., Chahla J. (2020). Perioperative Blood Flow Restriction Rehabilitation in Patients Undergoing ACL Reconstruction: A Systematic Review. Orthop. J. Sport. Med..

[19] Takarada Y., Takazawa H., Ishii N. (2000). Applications of vascular occlusion diminish disuse atrophy of knee extensor muscles. Med. Sci. Sports Exerc..

[20] Moher D., Liberati A., Tetzlaff J., Altman D.G. (2009). Preferred reporting items for systematic reviews and meta-analyses: The PRISMA statement. BMJ.

[21] Higgins J.P.T., Thomas J., Chandler J., Cumpston M., Li T., Page M.J., Welch V.A. (2020). Cochrane Handbook for Systematic Reviews of Interventions.

[22] Maher C.G., Sherrington C., Herbert R.D., Moseley A.M., Elkins M. (2003). Reliability of the PEDro Scale for Rating Quality of Randomized Controlled Trials. Phys. Ther..

[23] Herbert R., Moseley A., Sherrington C., Maher C. (2000). Physiotherapy Evidence Database. Physiotherapy.

[24] Sterne J.A.C., Savović J., Page M.J., Elbers R.G., Blencowe N.S., Boutron I., Cates C.J., Cheng H.-Y., Corbett M.S., Eldridge S.M. (2019). RoB 2: A revised tool for assessing risk of bias in randomised trials. BMJ.

[25] Ferraz R.B., Gualano B., Rodrigues R., Kurimori C.O., Fuller R., Lima F.R., De Sá-Pinto A.L., Roschel H. (2018). Benefits of Resistance Training with Blood Flow Restriction in Knee Osteoarthritis. Med. Sci. Sports Exerc..

[26] Harper S., Roberts L., Layne A., Jaeger B., Gardner A., Sibille K., Wu S., Vincent K., Fillingim R., Manini T. (2019). Blood-Flow Restriction Resistance Exercise for Older Adults with Knee Osteoarthritis: A Pilot Randomized Clinical Trial. J. Clin. Med..

[27] Segal N., Davis M.D., Mikesky A.E. (2015). Efficacy of Blood Flow-Restricted Low-Load Resistance Training for Quadriceps Strengthening in Men at Risk of Symptomatic Knee Osteoarthritis. Geriatr. Orthop. Surg. Rehabil..

[28] Segal N.A., Williams G.N., Davis M.C., Wallace R.B., Mikesky A.E. (2015). Efficacy of Blood Flow-Restricted, Low-Load Resistance Training in Women with Risk Factors for Symptomatic Knee Osteoarthritis. PM&R.

[29] Bryk F.F., dos Reis A.C., Fingerhut D., Araujo T., Schutzer M., De Paula Leite Cury R., Duarte A., Fukuda T.Y. (2016). Exercises with partial vascular occlusion in patients with knee osteoarthritis: A randomized clinical trial. Knee Surg. Sport. Traumatol. Arthrosc..

[30] Curran M.T., Bedi A., Mendias C.L., Wojtys E.M., Kujawa M.V., Palmieri-Smith R.M. (2020). Blood Flow Restriction Training Applied with High-Intensity Exercise Does Not Improve Quadriceps Muscle Function after Anterior Cruciate Ligament Reconstruction: A Randomized Controlled Trial. Am. J. Sports Med..

[31] Iversen E., Røstad V., Larmo A. (2016). Intermittent blood flow restriction does not reduce atrophy following anterior cruciate ligament reconstruction. J. Sport Health Sci..

[32] Ohta H., Kurosawa H., Ikeda H., Iwase Y., Satou N., Nakamura S. (2003). Low-load resistance muscular training with moderate restriction of blood flow after anterior cruciate ligament reconstruction. Acta Orthop. Scand..

[33] Hughes L., Paton B., Haddad F., Rosenblatt B., Gissane C., Patterson S.D. (2018). Comparison of the acute perceptual and blood pressure response to heavy load and light load blood flow restriction resistance exercise in anterior cruciate ligament reconstruction patients and non-injured populations. Phys. Ther. Sport.

[34] Hughes L., Patterson S.D., Haddad F., Rosenblatt B., Gissane C., McCarthy D., Clarke T., Ferris G., Dawes J., Paton B. (2019). Examination of the comfort and pain experienced with blood flow restriction training during post-surgery rehabilitation of anterior cruciate ligament reconstruction patients: A UK National Health Service trial. Phys. Ther. Sport.

[35] Slysz J., Stultz J., Burr J.F. (2016). The efficacy of blood flow restricted exercise: A systematic review & meta-analysis. J. Sci. Med. Sport.

[36] Lixandrão M.E., Ugrinowitsch C., Laurentino G., Libardi C.A., Aihara A.Y., Cardoso F.N., Tricoli V., Roschel H. (2015). Effects of exercise intensity and occlusion pressure after 12 weeks of resistance training with blood-flow restriction. Eur. J. Appl. Physiol..

[37] Yasuda T., Ogasawara R., Sakamaki M., Ozaki H., Sato Y., Abe T. (2011). Combined effects of low-intensity blood flow restriction training and high-intensity resistance training on muscle strength and size. Eur. J. Appl. Physiol..

[38] Fransen M., McConnell S., Harmer A.R., Van der Esch M., Simic M., Bennell K.L. (2015). Exercise for osteoarthritis of the knee. Cochrane Database Syst. Rev..

[39] Zampogna B., Papalia R., Papalia G.F., Campi S., Vasta S., Vorini F., Fossati C., Torre G., Denaro V. (2020). The Role of Physical Activity as Conservative Treatment for Hip and Knee Osteoarthritis in Older People: A Systematic Review and Meta-Analysis. J. Clin. Med..

[40] Zwart A., Dekker J., Lems W., Roorda L., Esch M., Leeden M. (2018). Factors associated with upper leg muscle strength in knee osteoarthritis: A scoping review. J. Rehabil. Med..

[41] Palmieri-Smith R.M., Thomas A.C., Wojtys E.M. (2008). Maximizing Quadriceps Strength after ACL Reconstruction. Clin. Sports Med..

[42] Loenneke J.P., Fahs C.A., Rossow L.M., Sherk V.D., Thiebaud R.S., Abe T., Bemben D.A., Bemben M.G. (2012). Effects of cuff width on arterial occlusion: Implications for blood flow restricted exercise. Eur. J. Appl. Physiol..

[43] Loenneke J.P., Allen K.M., Mouser J.G., Thiebaud R.S., Kim D., Abe T., Bemben M.G. (2015). Blood flow restriction in the upper and lower limbs is predicted by limb circumference and systolic blood pressure. Eur. J. Appl. Physiol..

[44] Hughes L., Jeffries O., Waldron M., Rosenblatt B., Gissane C., Paton B., Patterson S.D. (2018). Influence and reliability of lower-limb arterial occlusion pressure at different body positions. PeerJ.

